# Optimization of expression, purification and secretion of functional recombinant human growth hormone in *Escherichia coli* using modified staphylococcal protein a signal peptide

**DOI:** 10.1186/s12896-021-00701-x

**Published:** 2021-08-16

**Authors:** Garshasb Rigi, Amin Rostami, Habib Ghomi, Gholamreza Ahmadian, Vasiqe Sadat Mirbagheri, Meisam Jeiranikhameneh, Majid Vahed, Sahel Rahimi

**Affiliations:** 1 Department of Genetics, Faculty of Basic Science, Shahrekord University, P. O. Box 115, Shahrekord, 881 863 4141 Iran; 2 Department of Industrial Biotechnology, Research Institute of Biotechnology, Shahrekord University, Shahrekord, Iran; 3 Department of Industrial and Environmental Biotechnology, National Institute of Genetic Engineering and Biotechnology (NIGEB), Tehran, Iran; 4 Fisheries products processing group, Faculty of Fisheries and Environmental Sciences, Gorgan University of Agricultural Sciences and Natural Resources, Gorgan, Iran; 5 Pharmaceutical Sciences Research Center, Shahid Beheshti University of Medical Sciences, Niayesh Highway, Valiasr Ave, Tehran, Iran; 6 Department of Toxico/Pharmacology, School of Pharmacy, Shahid Beheshti, University of Medical Sciences, Niayesh Highway, Valiasr Ave, Tehran, Iran

**Keywords:** Sec pathway, Recombinant hGH, SpA signal peptide, Secretion

## Abstract

**Background:**

Human Growth Hormone (hGH) is a glycoprotein released from the pituitary gland. Due to the wide range of effects in humans, any disruption in hGH secretion could have serious consequences. This highlights the clinical importance of hGH production in the treatment of different diseases associated with a deficiency of this hormone. The production of recombinant mature hormone in suitable hosts and secretion of this therapeutic protein into the extracellular space can be considered as one of the best cost-effective approaches not only to obtain the active form of the protein but also endotoxin-free preparation. Since the natural growth hormone signal peptide is of eukaryotic origin and is not detectable by any of the *Escherichia coli* secretory systems, including Sec and Tat, and is therefore unable to secrete hGH in the prokaryotic systems, designing a new and efficient signal peptide is essential to direct hGh to the extracellular space.

**Results:**

In this study, using a combination of the bioinformatics design and molecular genetics, the protein A signal peptide from *Staphylococcus aureus* was modified, redesigned and then fused to the mature hGH coding region. The recombinant hGH was then expressed in *E. coli* and successfully secreted to the medium through the Sec pathway. Secretion of the hGH into the medium was verified using SDS-PAGE and western blot analysis. Recombinant hGH was then expressed in *E. coli* and successfully secreted into cell culture medium via the Sec pathway. The secretion of hGH into the extracellular medium was confirmed by SDS-PAGE and Western blot analysis. Furthermore, the addition of glycine was shown to improve hGH secretion onto the culture medium. Equations for determining the optimal conditions were also determined. Functional hGH analysis using an ELISA-based method confirmed that the ratio of the active form of secreted hGH to the inactive form in the periplasm is higher than this ratio in the cytoplasm.

**Conclusions:**

Since the native signal protein peptide of *S. aureus* protein A was not able to deliver hGH to the extracellular space, it was modified using bioinformatics tools and fused to the n-terminal region of hGh to show that the redesigned signal peptide was functional.

**Supplementary Information:**

The online version contains supplementary material available at 10.1186/s12896-021-00701-x.

## Introduction

Human Growth Hormone (hGH) or somatotropin is a glycoprotein which is released from the pituitary gland. Various forms of this hormone is available while the 191 amino acid length is more predominant [1]. hGH, as a multifunctional hormone, plays various roles in cells. For example, it can have inhibitory effects on glycolysis followed by a direct effect on protein synthesis [2], as well as increase the absorption and retention of calcium, magnesium and phosphate ions in the body. Any disorder in the secretion of hGH in childhood or adolescence can lead to a variety of diseases including gigantism, acromegaly, and dwarf diseases [2, 3]. hGH has been used in clinics since 1985 to treat a variety of children as well as adults hGH-related disorders, including Prader-Willi syndrome, chronic renal insufficiency, Turner syndrome, AIDS-related wasting, fat accumulation associated with lipodystrophy in adults [4, 5]. It may also be associated with some metabolic complications and even mellitus diabetes [6]. On the other hand, recombinant therapeutical proteins have received a great deal of attention in recent years due to their advantages including low side effects, minimized cytotoxicity, high selectivity, and very low non-specific interactions [7, 8]. These products must be physically and chemically stable enough. Physical instability refers to changes that occur in a three-dimensional structure [9], while chemical instability refers to any chemical changes in a protein that involves the formation of a bond resulting in the formation of a new chemical [10]. Proper folding of therapeutic proteins is essential for their function [10]. Recent advances in genetic engineering have made it possible for microorganisms to be used for expression of heterologous proteins.

In this approach, heterologous proteins are expressed either as cytoplasmic or intracellularly or secreted as extracellularly [11]. In the first method, a methionine amino acid is necessarily added to the amino terminus as the starting codon for protein expression. Given that this methionine has been shown to stimulate the immune system against heterologous proteins, and since this is very important for pharmaceutical proteins, additional methods must be used to remove this methionine such as the use of different peptidases which imposes an additional step on the system [12]. Expression of the intercellular form can lead to the formation of inclusion bodies that has no functions [13]. Secretion of the heterologous proteins into the extracellular medium is an appropriate choice for obtaining the active, endotoxin-free form of the proteins that can be achieved by fusing the signal peptides to the N-terminal region of the protein. Signal peptides play a vital role in directing the target protein into the periplasmic and extracellular medium [14]. The secretion of protein into the growth medium has many advantages over intracellular expression. These benefits include reducing production costs, facilitation of downstream processes, and stability of the expressed proteins and originality of their N-terminal sequence [14]. In addition, the secretion of heterologous protein in *E. coli* is an effective strategy to help reduce the contamination with host cell proteins and thus improve the quality of the recombinant products [11]. In addition, if the product is secreted into the culture medium, cell lysis is no longer necessary to recover the product and the product is obtained from the culture medium without disrupting the cell to release cytoplasmic protein contaminants [12]. This strategy is also preferred because it greatly reduces proteolytic activity in the culture medium that occurs immediately after cell lysis with the release of proteases. Various strategies have been used to increase the extracellular secretion of hGH in *E. coli* strain BL21(DE3), including the use of physical and chemical methods such as osmotic shock, freeze-thaw cycles, lysozyme treatment, and chloroform shock, each of which has its own problems. In the present study, the recombinant hGH was expressed and purified with a very low level of endotoxin contamination and without N-terminal methionine in *E. coli* [12]. For this purpose, signal peptide from *Staphylococcus aureus* protein A (SpA) was modified, redesigned based on the Sec secretory system and linked to the coding region of the mature form of the HGH. Finally, optimization of expression and secretion was carried out using Response Surface Methodology (RSM).

## Results

### Optimization of codon usage

**Fig. 1:**
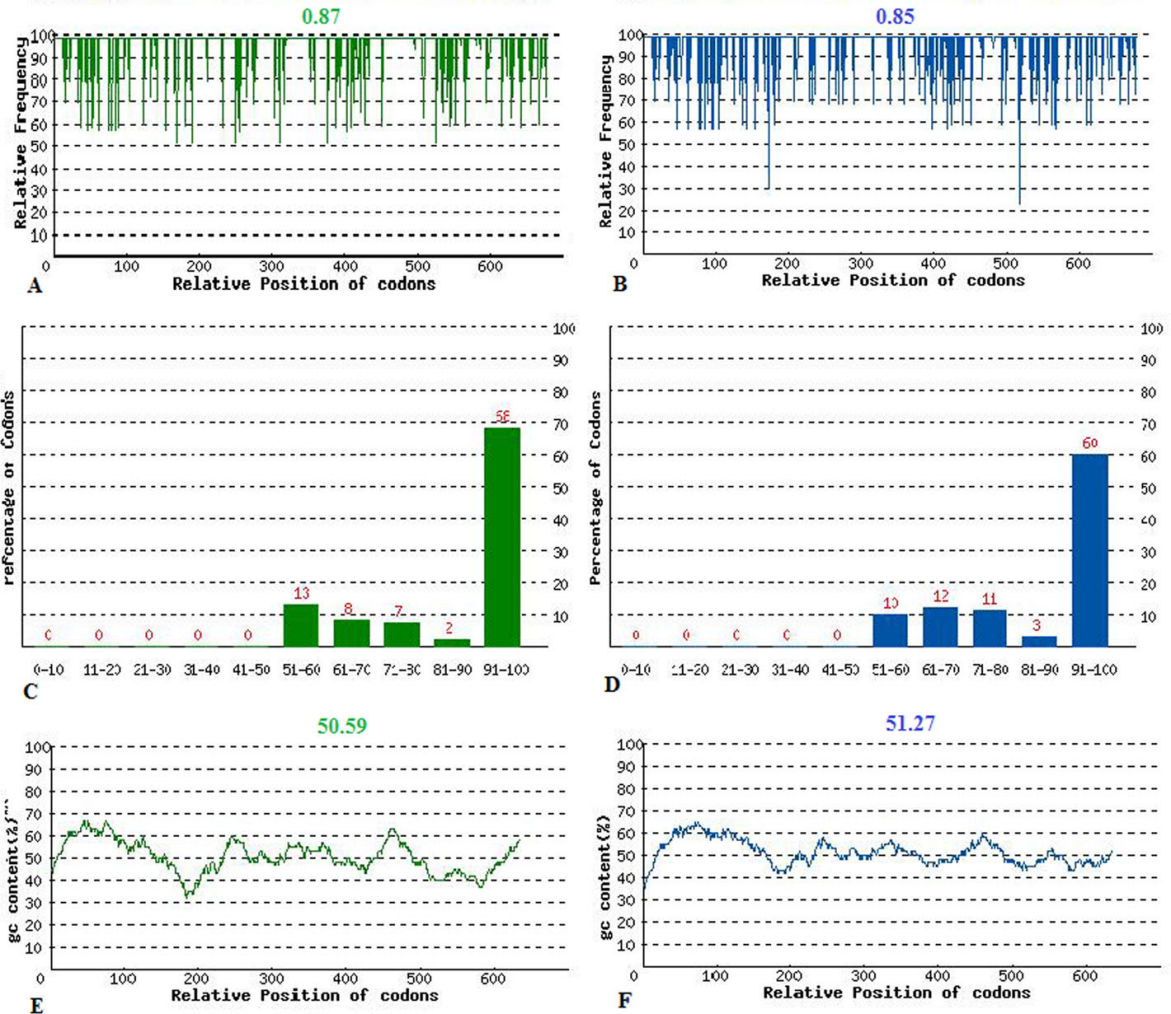
The extension of codon usage frequency throughout the codon optimized gene sequence, CAI; after optimization (**A**) and before optimization (**B**). The FOP; after optimization (**C**) and before optimization (**D**). Average GC content; after optimization (**E**) and before optimization (**F**)

The sequence of optimized hGH gene was deposited in GenBank with the accession numbers of MT321110. Rare gene codons that could reduce translation efficiency were adapted to the expression system of *E. coli* codon usage. The Codon Adaption Index (CAI) was improved from 0.85 to 0.87. Meanwhile, GC content was also optimized to increase the length of the mRNA half-life and its stability, and the stem-loop structures that blocked the ribosomal connection were removed (Fig. 1 A and Fig. 1 B). FOP of 68 was attained after optimization (Fig.1 C and Fig. 1 D). The ideal percentage range of GC content is between 30 and 70%. GC content adjustment resulted in the average of 50.59% after optimization (Fig. 1 E and Fig. 1 F).

### Design of secretory signal peptide

**Fig. 2:**
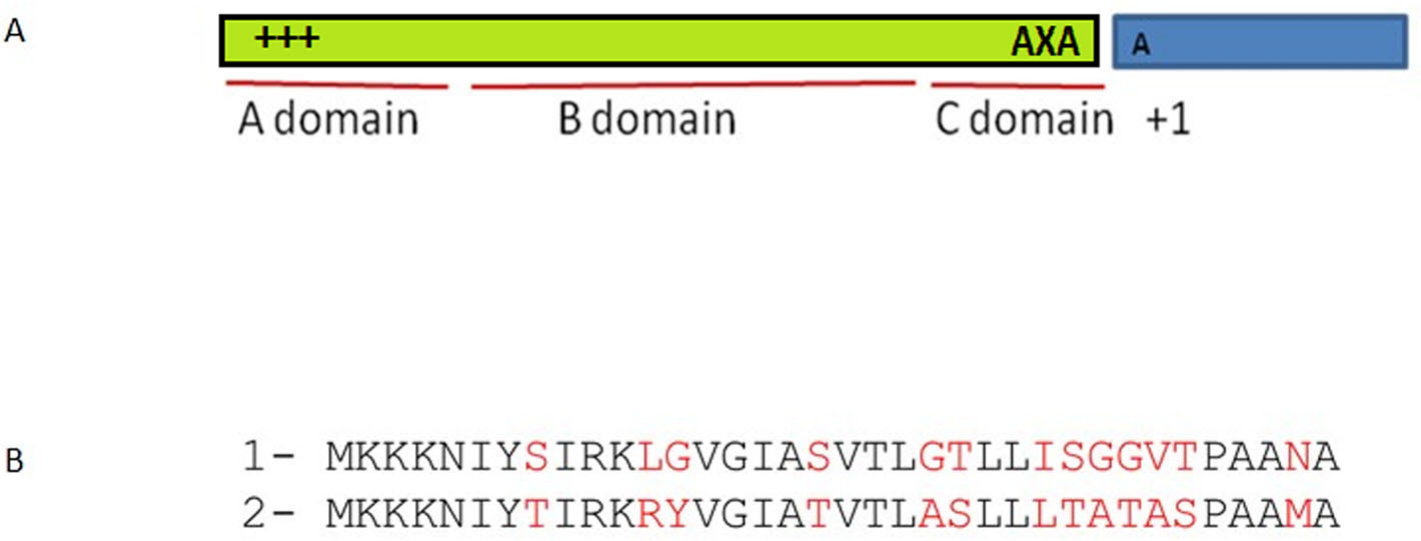
General structure of the natural sec-dependent signal sequences and alignment of the native and modified signal peptide for secretion of hGH. Panel **A** is a schematic of the *S. aureus* signal peptide showing the Ala-X-Ala box consensus that is a peptidase cleavage site. This panel also shows the presence of different domains in the sec-dependent signal sequences. Panel (**B**) Shows alignment and comparison of the amino acid sequence of natural secretive signal (No. 1) and the amino acid sequence of the modified secretive signal (No. 2)

Figure 2, Panel A is a schematic of the *S. aureus* signal peptide showing the consensus sequence of the Ala-X-Ala box as well as the three associated domains. The C-terminal of the signal peptide contains the signal peptidase cleavage site, which has a conserved “(−3, −1) amino acids” often as Ala-X-Ala [12].. The designed secretory signal peptide includes three domains. Domain A consists of a region with positively charged amino acids. Domain B consists of a hydrophobic region with a stretch of hydrophobic amino acids and domain C consists of the conserved site for signal peptidase cleavage. The sequence of the natural signal and the designed signal based on the general structure of the natural sec-dependent signal sequences are shown in Fig. 2. The altered amino acids have a lower line, and the alignment of these two signals shows their amino acid different.

Our initial studies showed that the natural signal peptide of the protein A is unable to secrete hGH. Therefore, the natural secretory signal was modified and optimized. Our results show that comparing to the natural signal peptide, the modified one became functional in secreting the hGH. The modified signal peptide is able to secrete the recombinant hGH to the both periplasm space and culture medium. As can be seen in Fig. 2, panel B, a series of amino acid changes have taken place in different regions of the peptide signal, including the N, H, and C2 regions, which increased the ability of the altered peptide signal to secrete growth hormone out of the cell.

For example, replacing arginine with leucine in the modified form of signal peptide has given it a more positive charge in the N domain and increased its ability to enter the cell membrane which has a negative charge. Another important change is seen in the H domain, in which the amino acid isoleucine replaces threonine and the amino acid serine is changed to alanine in the hydrophobic domain, which, as shown in Fig. 5, increases the hydrophobicity of the H region.

### Designed gene and tertiary structure

**Fig. 3:**
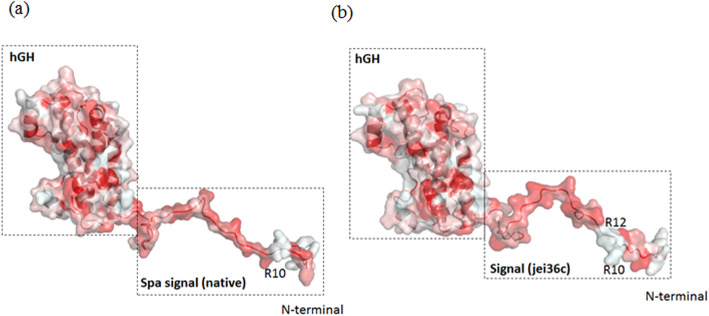
The tertiary structure of hGH-mutant (jei36c) have two Arginine residues R10 and R12 but hGH native have a one Argentine residue R10

The schematic view of the pET26 plasmid as well as the designed gene structure is shown in supplementary data file 1. The signal peptide hGH-mutant (jei36c) has two Arginine residues R10 and R12 while the signal peptide of the native hGH has one Argentine residue R10. (Fig. 3).

### Signal peptide functional analysis

**Fig. 4:**
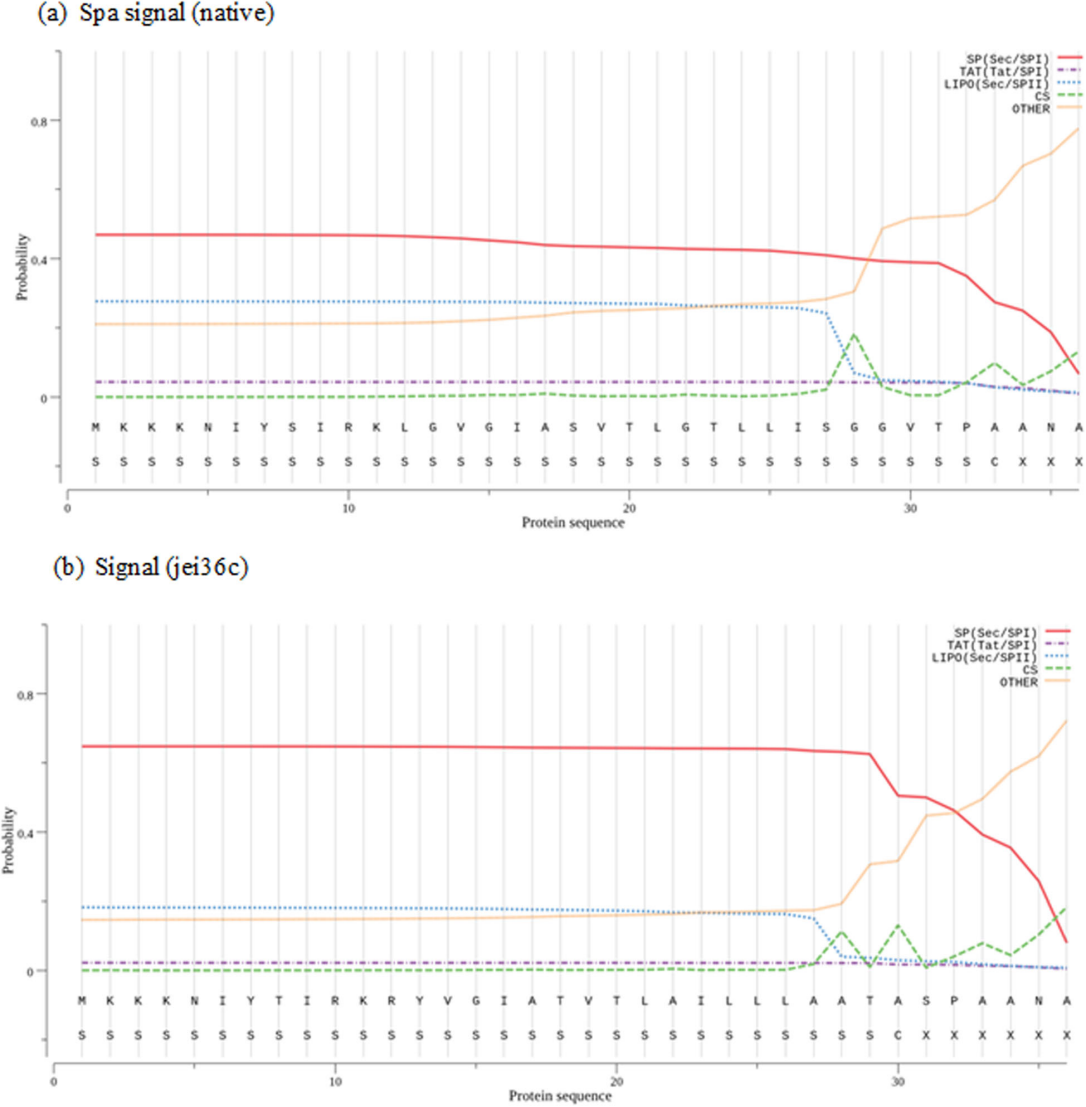
Signal peptide prediction. (**a**) Spa signal (native), (**b**) Signal (jei36c). Sec/SPI: standard” secretory signal peptides transported by the Sec translocon and cleaved by Signal Peptidase I; Tat signal peptides (Tat/SPI), which direct their proteins through a twin-arginine translocation; Sec/SPII: lipoprotein signal peptides transported by the Sec translocon and cleaved by Signal Peptidase II

The results of likelihood score-based predictions for the native and modified jei36c signal peptides are shown in Table 1. For prediction with signal peptide (SP) and tail anchor (TA) datasets, scores were calculated from the probability values of TA and SP models (Table 1 and Fig. 4).

**Table 1:** Signal peptide prediction

Protein type	Signal peptide (Sec/SPI)	TAT signal peptide (Tat/SPI)	Lipoprotein signal peptide (Sec/SPII)	Other
Spa signal (native)	0.47	0.04	0.27	0.21
Signal (jei36c)	0.64	0.02	0.18	0.14

### Hydrophobicity characteristics of the designed signal peptide

**Fig. 5:**
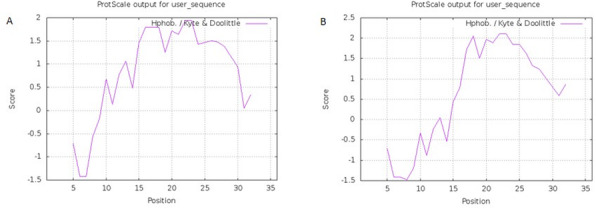
The graphs show the hydrophobicity areas of the natural (panel **A**) and modified (panel **B**) signal peptide using the ProtScale software. The horizontal axis shows amino acids position and the vertical axis shows the degree of hydrophobicity. The numbers above zero (positive) are hydrophobic regions and the numbers below zero (negative) are hydrophilic regions of the signal peptide. After modification, the hydrophobicity of the signal peptide as well as number of hydrophobic amino acids increased

ProtScale online software was used to evaluate the hydrophobicity of the designed signal peptide comparing to the native signal peptide [15] (Fig. 5). Drawing the hydrophobicity and hydrophilicity profiles for these two signal peptides and examining their different domains to observe the changes in their hydrophobicity and hydrophilicity is shown in Fig. 5 (panels E and B). Comparison of these changes shows that the corresponding profiles in these two signal peptides have changed significantly.

The changes in the mutant signal peptide have been able to increase its hydrophilicity of the N region and, conversely, the hydrophobicity of the H region, and ultimately facilitate the secretion of hGH out of the cell. Taken together, these changes increasing the efficiency of this signal peptide in secreting hGH and make it optimized for the secretion of growth hormone from the sec pathway.

### Stability of mRNA

**Fig. 6:**
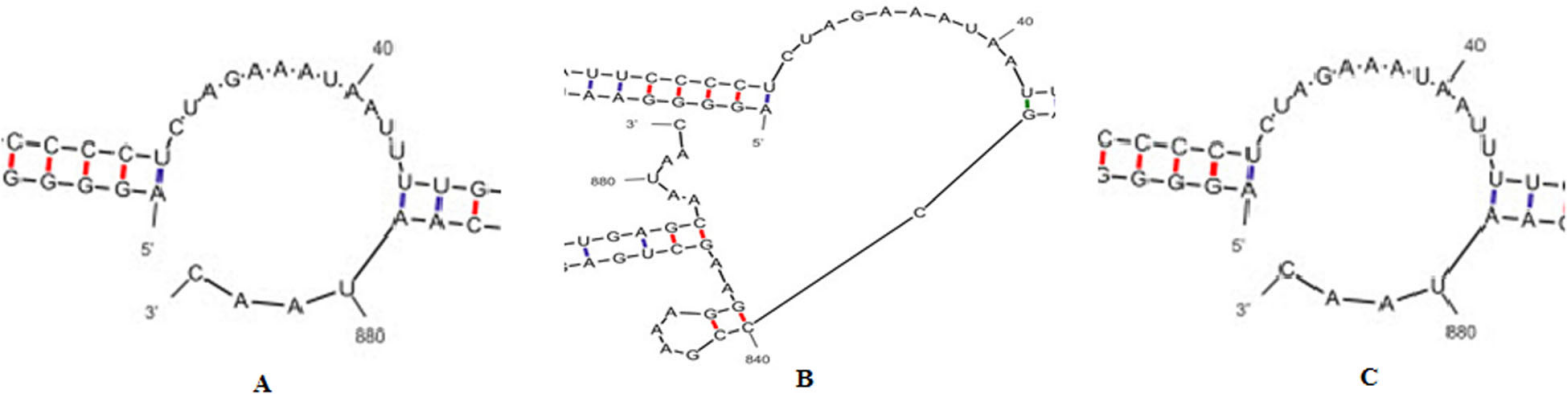
Analysis of mRNA stability using mfold online server. Initial region related to the second structure of the different mRNAs encoding the growth hormone with different designed signal peptides

To evaluate the stability of the transcribed mRNAs, the mRNA sequences of hGH resulted from fusion of both native and designed signal peptides were submitted to the mfold online server (http://unafold.rna.albany.edu/?q=mfold/RNA-Folding-Form) [16]. During codon optimization, an attempt was made to remove the secondary structure that attenuates or stops mRNA translation, and as it is shown in the Fig. 6, the translation inhibitory structure is not seen in the starting region.

### Evaluation of recombinant growth hormone expression

Different pET26-based plasmids containing the fusion of various signal peptides to the hGH coding region were transformed into *E. coli* BL21 (DE3) strain.

Recombinant growth hormone expression was induced under different temperatures as well as various concentrations of glycine and lactose. The following are the optimum conditions which led to the high level of hGH expression in our experiment.

**Fig. 7:**
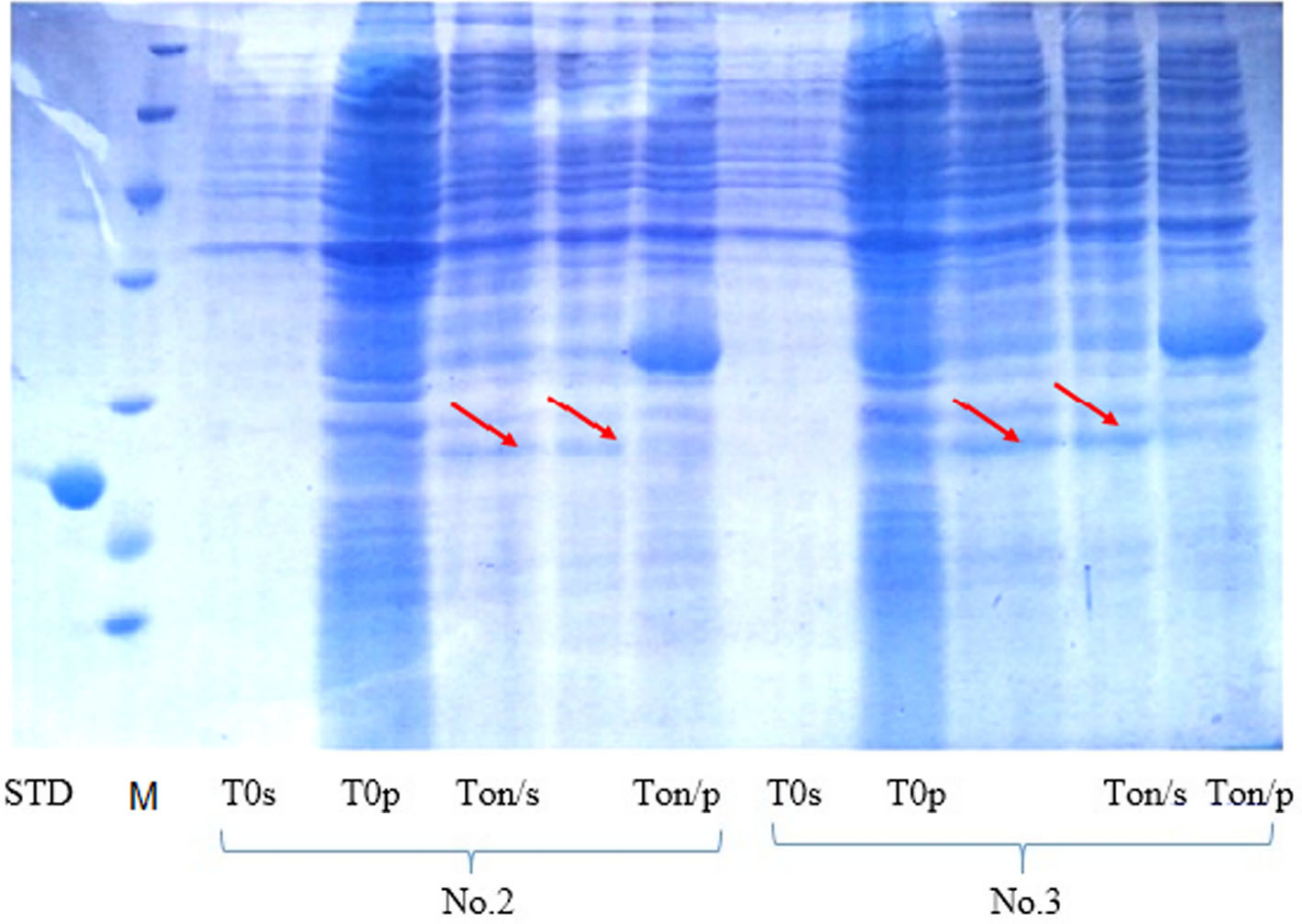
Evaluation of the recombinant hGH expression extracted from cytoplasm and supernatant. hGH expression band appears in the supernatant of the bacterial colonies related to the signal peptides 2 and 3. T0s stands for supernatant of culture medium at T0, T0p is bacterial pellet protein content at T0 and To/n stands for T overnight. STD is a commercially available hGH from Novo Nordisk company which was used as a positive control

Test conditions A: induction of expression with 10% lactose, 1.5% glycine and induction in OD 1.5, incubation at 30 °C overnight. The growth hormone bands are shown in the Fig. 7 and supplementary data file 2.

**Fig. 8:**
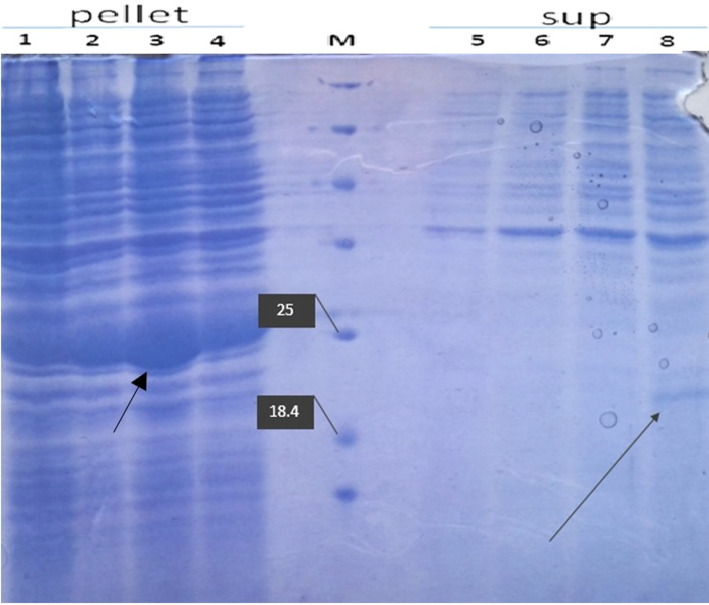
Evaluation of the recombinant hGH expression derived from cytoplasmic and supernatant fraction of *E. coli* culture. Lines 1 to 3 are total protein extracted from various colonies, line 4 is related to the total protein extracted from various colonies related to the modified hormone variable, 5 to 7, are related to the protein derived from the supernatant solution of various colonies related to the growth hormone having a natural signal, and the line 8 of the protein derived from the supernatant solution of the various colonies related to the modified growth hormone and M is the protein molecular weight marker

Test condition B includes induction of expression with 7% lactose, glycine 1% after reaching the bacterial OD to 1.5 and incubation at 25 °C. In this experiment, the sampling time was also reduced to eight hours. In this situation, the supernatant from the bacterial culture was centrifuged at 11000×g for 5 min and the supernatant was precipitated by TCA. As shown in the Fig. 8 and supplementary data file 3, the hGH bands are present in all supernatants except No. 3.

**Fig. 9:**
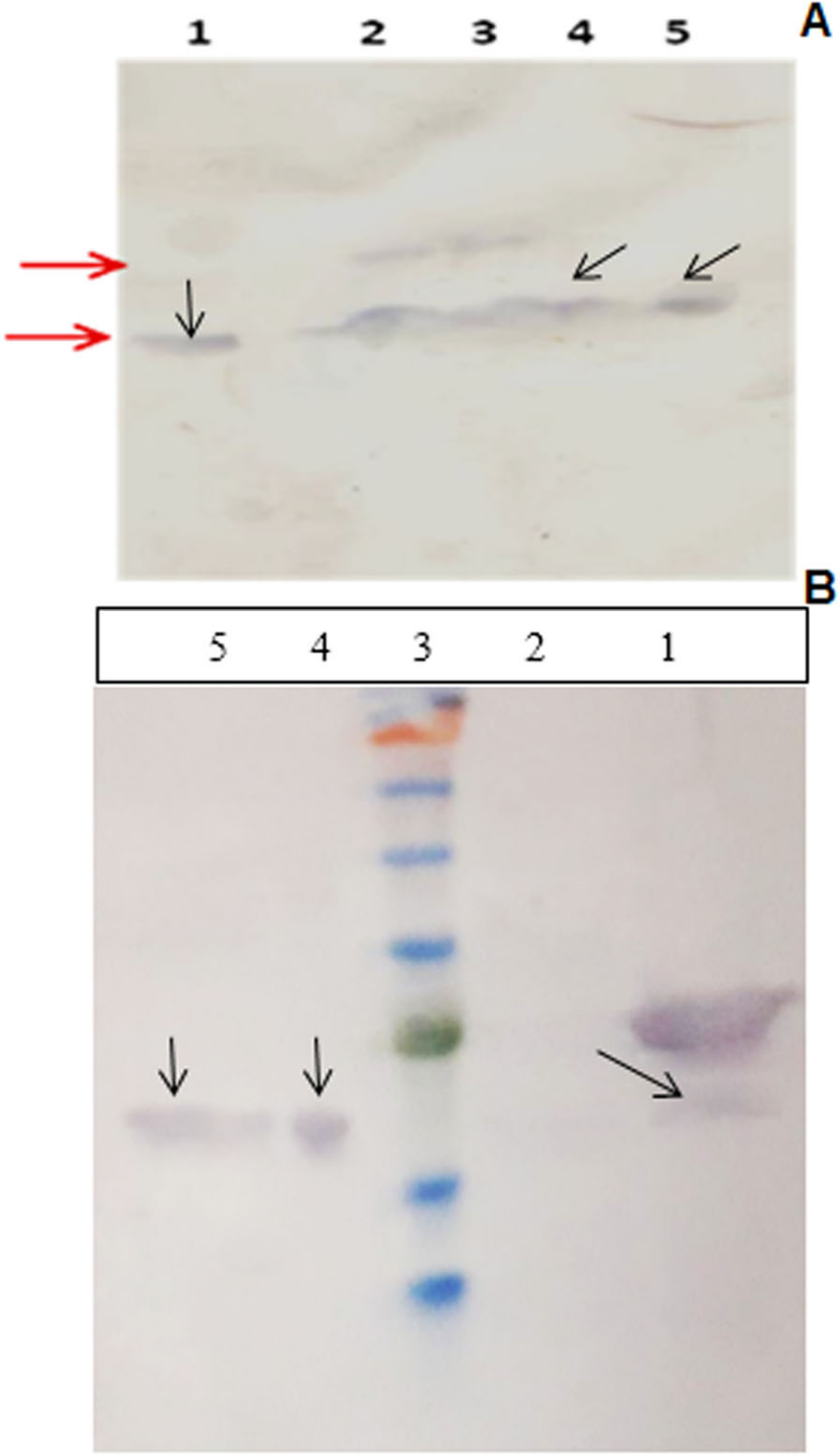
Panel **A**: Western blot analysis of protein samples extracted from cytoplasm (columns 2 and 3), periplasm (column 4), and culture medium (column 5). Column 1 is the standard processed form of hGH (a commercially available hGH from Novo Nordisk company which was used as a positive control). Panel **B**: Western blot analysis of different protein fractions obtained after expression of hGH from recombinant *E. coli*. The lanes are loaded as follows: lane 1, cytoplasmic fraction, lane 2, protein extracted from cytoplasm before induction as a negative control; lane 3 is a molecular weight protein marker; lane 4, a commercially available hGH from Novo Nordisk company which was used as a positive control and lane 5 is the periplasmic fraction of hGH obtained by osmotic shock preparation. Both panels (panel **A** and panel **B**) were run at the same time and only in the gel displayed in panel B protein marker was loaded which can be easily detect the hGH bands. In addition, in both panels (lane 1 the panel A and lane 4 in panel B), are a commercial product of the hGH from Novo Nordisk company which was used as a control, which can also be considered as a marker. This standard hGH has the same mobility on the gel as our processed form of the recombinant

The expression of recombinant hGH was confirmed by western blotting. As shown in Fig. 9A and B and supplementary data file 4, Western blot analysis using a specific polyclonal Antibody raised against hGH was used to confirm the expression of the hormone. Arrows in all Figs. 6, 7 and 8 represent the expressed recombinant hGH that is processed by the cell secretory system and secreted into the environment.

### Protein purification

**Fig. 10:**
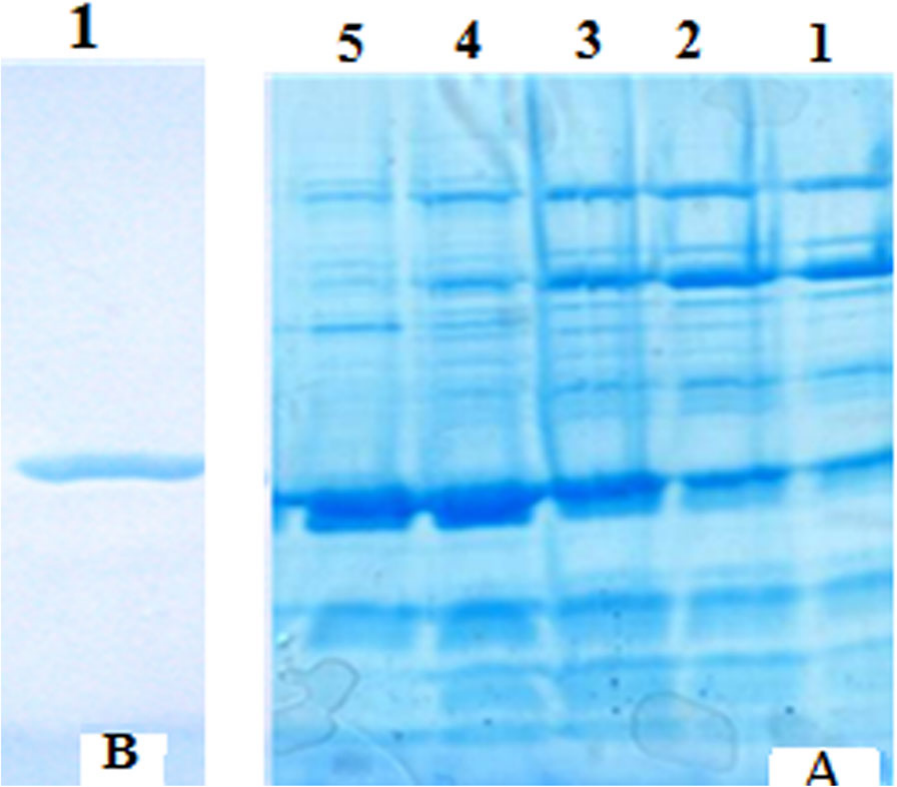
Purification of hHG using ion exchange chromatography and SDS-PAGE analysis. Panel **A**: Columns 1 to 5, hGH before purification. Panel **B**: Column 1 shows the final purified protein by affinity chromatography after the final cleansing of the hGH through the use of salt., the figure was cropped and grouped from different part of the different gels and was made explicit using white space between panel A and panel B

The expressed recombinant hGH was then purified by affinity chromatography and purification was evaluated using SDS-PAGE analysis. As shown in Fig. 10, the hGH is shown as a single, pure band (Line 6, panel B), comparing to its pre-purification state (Lines 1–5, panel A).

### Optimization results of the periplasmic hGH expression and secretion

In order to investigate the possible interactions between the factors affecting the production of periplasmic and cytoplasmic hGh production along with their optimal levels, the central composite design (CCD) was used. The matrix is designed and the responses are shown in Table 2. Analysis of variance (ANOVA) based on the response surface model for the periplasmic hGH (Table 3) was calculated. Regarding the expression of periplasmic hGH,, the Model F value of 3.23 implies that the model is significant. Values of “Prob > F” less than 0.05, indicate the significance of the model terms. In this case, C: Cell density at induction time (OD600), and D: Post induction time (h) are significant. Values > 0.1 demonstrate that the model terms are not significant. The “Lack of Fit F value” of 1.42 implies that relative to the pure error, Lack of Fit is not significant. We prefer the model to fit and it is in suitable agreement (Table 3).

**Table 2:** Variables are showing observed values of periplasmic and cytoplasmic hGH hormone expression

Run	IPTG (mM)	Temperature ( ˚C)	Cell density at induction time (OD_**600**_)	Post induction time (H)	Cytoplasmic Production (ug/ml)	Periplasmic Production (ug/ml)
1	1	20	1.5	16	42	60
2	0.2	33	1.5	16	20	60
3	0.2	20	0.5	4	0	0
4	0.6	26.5	1	4	0	0
5	0.6	26.5	0.5	10	0	0
6	1.3	26.5	1	10	20	0
7	1	33	1.5	4	19	90
8	1	33	0.5	4	0	0
9	0.2	26.5	1	10	32	30
10	0.6	26.5	1.5	10	33	60
11	0.6	20	1	10	9	26
12	0.6	26.5	1	10	35	100
13	1	20	0.5	16	19	0
14	0.6	26.5	1	16	46	84
15	0.6	26.5	1	10	44	95
16	0.6	26.5	1	10	45	110
17	0.2	33	0.5	16	60	147
18	0.2	20	1.5	4	58	54
19	0.6	33	1	10	39	76
20	0.6	26.5	1	10	28	34
21	0.6	26.5	1	10	16	26

**Table 3:** Analysis of variance (ANOVA) of the response surface model for the periplasmic hGH hormone production

Source	Sum of Squares	df	Mean Square	F Value	P-value	Prob>F
Model	263.58	10	26.36	3.23	0.0393	Significant
A	23.93	1	23.93	2.93	0.1178	
B	6.55	1	6.55	0.80	0.3918	
C	78.11	1	78.11	9.56	0.0114	
D	42.00	1	42.00	5.14	0.0468	
AB	16.74	1	16.74	2.05	0.1828	
AC	25.43	1	25.43	3.11	0.1082	
AD	1.129	1	1.129	1.39	0.9909	
BC	12.47	1	12.47	1.53	0.2451	
BD	3.78	1	3.78	0.46	0.5119	
CD	22.67	1	22.67	2.72	0.1268	
Residual	81.73	10	8.17			
Lack of fit	55.64	6	9.27	1.42	0.3824	Not Significant
Pure Error	26.09	4	6.52			
Cor Total	345.31	20				
Std.Dev.	Mean	R-Squared				
2.86	5.80	0.76				

A: IPTG (mM), B: Temperature (˚C), C: Cell density at induction time (OD_600_), D: Post induction time (h)

Multiple regression analysis of the data was performed, and a first-order polynomial equation for the periplasmic hGH (ug/mL) (Y) according to the coded factors was expressed in eq. 1:

1 $$ \mathrm{Y}=5.89-2.44\ \mathrm{A}+1.81\ \mathrm{B}+2.79\ \mathrm{C}+4.58\ \mathrm{D}+3.23\ \mathrm{A}\mathrm{B}+1.78\ \mathrm{A}\mathrm{C}+0.027\ \mathrm{A}\mathrm{D}-1.25\ \mathrm{B}\mathrm{C}-1.19\ \mathrm{B}\mathrm{D}-1.68\ \mathrm{C}\mathrm{D} $$

A, B, C, and D, are coded values for IPTG (mM), temperature (°C), cell density at induction time (OD_600_) and post induction time (h), respectively. The above equation for the actual variables can be rewritten as eq. 2:

2 $$ \mathrm{Y}=-9.72-48.08\ \mathrm{A}+0.22\ \mathrm{B}+16.03\ \mathrm{C}+2.12\ \mathrm{D}+1.24\ \mathrm{A}\mathrm{B}+8.91\ \mathrm{A}\mathrm{C}+0.01\ \mathrm{A}\mathrm{D}-0.38\ \mathrm{B}\mathrm{C}-0.03\ \mathrm{B}\mathrm{D}-0.56\ \mathrm{D}\mathrm{C} $$

**Fig. 11:**
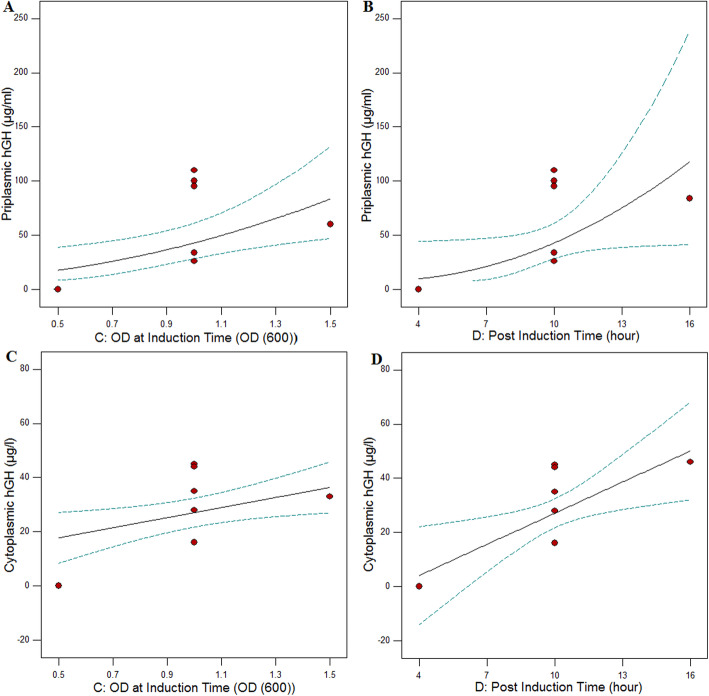
Significant single factor C; OD at induction time at IPTG = 0.6 mM, temperature = 26 °C and post induction time 10 h for periplasmic (**A**) and cytoplasmic (**C**) expressions. Significant one factor D; post induction time at induction time at IPTG = 0.6 mM, temperature = 26 °C and OD at induction time = 1 for periplasmic (**B**) and cytoplasmic (**D**) expressions

According to the Fig. 11, the optimum conditions for maximum expression of recombinant periplasmic hGH (panels, A and B) in *E. coli* are suggested as follows: IPTG = 0.6 mM, Temperature = 26 °C, post induction time = 10 h and OD at induction time = 1.

### Optimization results of the cytoplasmic hGH expression

The designed matrix and the responses are shown in Table 2. Analysis of variance (ANOVA) based on response surface model for cytoplasmic hGH (Table 4) was calculated. For cytoplasmic hGH expression, the Model F value of 4.92 implies that the model is significant. Values of “Prob > F” less than 0.05, show that the model terms are significant. In the case of cytoplasmic expression, C: Cell density at induction time (OD600), D: Post induction time (h) and interaction between B: Temperature (°C), and C: Cell density at induction time (OD600) (BC) are significant model terms. The “Lack of Fit F value” of 0.72 implies that Lack of Fit is not significant relative to the pure error. The “R Squared” of 0.83 is in reasonable agreement (Table 4).

**Table 4:** Analysis of variance (ANOVA) of the response surface model for the cytoplasmic hGH hormone production

Source	Sum of Squares	df	Mean Square	F-Value	P-value	Prob>F
Model	5905.05	10	5905.05	4.92	0.0095	Significant
A	73.12	1	73.12	0.61	0.4532	
B	450.00	1	450.00	3.75	0.0816	
C	864.90	1	864.90	7.21	0.0229	
D	1058.00	1	1058.00	8.81	0.0141	
AB	360.00	1	360.00	3.00	0.1140	
AC	72.00	1	72.00	0.60	0.4566	
AD	490.00	1	490.00	4.08	0.0709	
BC	1300.50	1	1300.50	10.83	0.0081	
BD	24.04	1	24.04	0.20	0.6641	
CD	1104.50	1	1104.50	9.20	0.0126	
Residual	1200.29	10	120.03			
Lack of fit	619.09	6	103.18	0.71	0.6635	Not Significant
Pure Error	581.20	4	145.30			
Cor Total	7105.81	20				
Std.Dev.	Mean	R-Squared				
10.96	26.90	0.83				

A: IPTG (mM), B: Temperature (˚C), C: Cell density at induction time (OD_600_), D: Post induction time (h)

Multiple regression analysis of the data was performed and a first-order polynomial equation for the cytoplasmic hGH (ug/mL) (Y) regarding coded factors was expressed in eq. 3:

3 $$ \mathrm{Y}=27.06-4.26\mathrm{A}+15\mathrm{B}+9.3\mathrm{C}+23\mathrm{D}+15\mathrm{AB}+3\mathrm{AC}+17.5\mathrm{AD}-12.75\mathrm{B}\mathrm{C}+2.99\mathrm{BD}-11.75\mathrm{CD} $$

where A, B, C, and D, are coded values for IPTG (mM), temperature (°C), cell density at induction time (OD_600_), and post induction time (h), respectively. In the actual variables, the above equation can be rewritten as eq. 4:

4 $$ \mathrm{Y}=-62.95-251.44\mathrm{A}+2\mathrm{B}+152.79\mathrm{C}+1.34\mathrm{D}+5.77\mathrm{AB}+15\mathrm{C}+7.29\mathrm{AD}-3.92\mathrm{B}\mathrm{C}+0.077\mathrm{BD}-3.92 $$

According to the Fig. 11, the optimum conditions for maximum cytoplasmic recombinant hGH expression (panels, C and D) in *E. coli* were suggested as IPTG = 0.6 mM, temperature = 26 °C, post induction time 10 h and OD at induction time = 1.

**Fig. 12:**
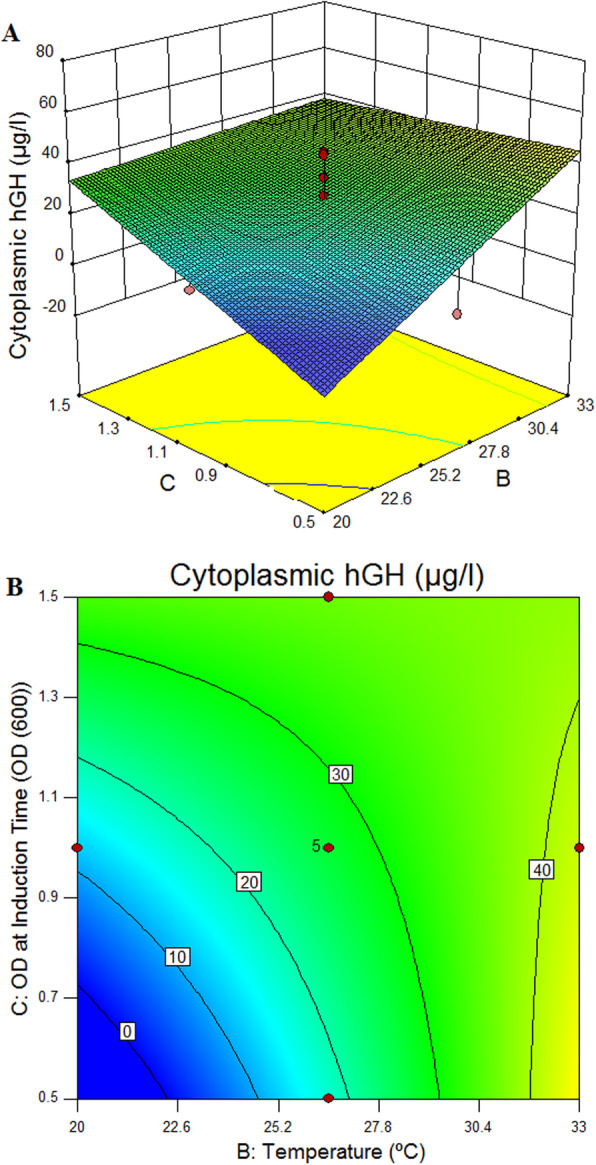
3D surface and contour of significant interaction between BC at IPTG = 0.6 mM and post induction time 10 h in cytoplasmic expression

Figure 12 shows the interactions between temperature and OD at induction time (OD_600_) at IPTG = 0.6 mM and post induction time 10 h for cytoplasmic hGH expression.

### Determination of functional hGH concentration

After generating the standard curves for each test by plotting the absorbance versus the concentration of each controls, concentrations of the active form of the hGH were obtained from the standard curve (all row data were reported in supplementary data file 5). The results showed that the amount of active form of hGH produced in the periplasm was about 28.12% of the total proteins, while this amount was equal to 10% for the cytoplasm fraction. These results indicate that the secretion of hGH into the periplasmic space by this method causes the protein to fold into its proper structural conformation.

## Discussion

Since its FDA approval in 1985, hGH has been used for clinical applications for more than three decades [17]. A variety of expression hosts has been used for production of hGH including mammalian, yeast and bacterial cells [18–20]. The use of *E. coli* as an expression host has several advantages including the possibility of easy manipulation of bacteria, low growth cost, rapid cell growth and the possibility of culturing high-density cells, which together make it an ideal system, especially from economic view compared to other expression systems [21]. Regarding the expression of recombinant hGH, various strategies have been used for protein expression, including the use of a wide range of different hosts, including eukaryotes such as mammalian cells, yeasts of *Saccharomyces cerevisiae*, *Pichia pastoris*, *Cluyveromyces lactis,* and prokaryotes such as *E. coli*. However, it can be mentioned with certainty that *E. coli* is one of the most widely used hosts for the production of pharmaceutical recombinant proteins on an industrial scale. This system is the good choice for the functional expression of non-glycosylated proteins. The advantages of this system include fast growth, high cell density using simple and low cost growth medium, known genetic information, availability of a large number of expression vectors and availability of mutant host strains, simple transfer, easy control, possibility of mass production of recombinant proteins and thus cost-effectiveness. In the case of yeast and mammalian hosts, in most cases, protein production is much lower and more expensive than *E. coli* host is. A search of the literature shows that in only one study without de novo design of a signal peptide, the hGH coding region was fused to the pelB signal sequence in the commercial pET26 vector and expressed in as a *E. coli* host and was able to direct hGH to the periplasm [22]. However, the level of expression was very low compared to our developed system. The secretion of recombinant hGH in the system developed by our research team in this study was much higher than the hGH production in that study.

During the cytoplasmic expression of recombinant proteins in *E. coli*, a single methionine is necessarily added to its N-terminal region. This may not be a problem for industrial enzymes, but in the case of recombinant proteins with pharmaceutical use, in addition to causing unwanted side effects, it is shown that it may stimulate the immune system and produce antibodies against these proteins [23]. Although this methionine can be removed using enzymatic reactions, its removal might impose some problems and additional costs on the system for their mass production, which is not desirable [24].

A major solution to overcome this problem in *E. coli* is the secretion of recombinant proteins into the culture medium using a suitable secretory signal peptide. Extracellular production of recombinant proteins has many advantages. The release of the recombinant proteins into the culture medium, in addition to reducing costs in the industry, eliminates the need to disrupt the host cell to extract and purify the proteins. As a result, proteases as well as endotoxins are not released into the environment. In addition, by continuously producing target proteins in the host, more recombinant proteins can be obtained in the fermenter. Although there are advantages to expressing proteins as periplasmic or cytoplasmic, each has disadvantages that limit the use of these methods, such as creating insoluble forms as inclusion body in the cytoplasm or obtaining small amounts of protein in the periplasmic method due to the limited capacity of the periplasm in the *E. coli*. Several classes of proteins, such as some toxins, are naturally secreted by *E. coli*, and others can enter the environment from the periplasmic space, possibly due to increased cell membrane permeability during the long incubation period [14]. In this study, the hGH was secreted into the medium through the type II secretion system named Sec pathway. The Sec-dependent system in gram negative bacteria is made of a channel called the SecYEG complex and a translocation protein known as SecA which has ATPase activity. Signal peptide sequences in the N-terminal region of proteins lead these proteins to the Sec system and eventually secrete them out of the cell [11]. These signal peptides have a three-dimensional structure with a positively charged N region, a hydrophobic H region, and a C region in which Ala-X-Ala motif is identified and cleaved by the cellular signal peptidase enzymes [11, 12].

The hGH is a eukaryotic protein and has its own native signal peptide that is recognized by the eukaryotic secretory pathway machinery. However, to secret a recombinant hGH in *E. coli* that is a prokaryotic host, this signal is not recognized to direct hGH onto the periplasm and then extracellular space. It is known that the secretion processes, mechanisms and components of eukaryotic and prokaryotic secretion systems are different. In this study, the native signal peptide of *S. aureus* protein A (SpA) was used first to direct the recombinant hGH onto the periplasm and to the extracellular part. Using the native signal peptide, we were able to direct its native partner protein, SpA, to extracellular and periplasmic fraction [25], in *E. coli* heterologous expression system. Nevertheless, the use of this signal peptide in this study was not functional and was not successful in directing the recombinant hGH out of the cell. Then the native peptide signal was redesigned and changes were made in its various domains and a novel signal peptide was designed using the relevant softwares. Fortunately, the changes were effective and yielded good results, as this new signal was detected by components of the bacterial secretory system and was able to direct recombinant hGH into periplasm and extracellular space in the *E. coli.* The natural signal sequence and the designed signal based on the general structure of the sec-dependent signal sequences including all three domains introduced above are shown in Figs. 2 and 3. The probabilities shown on the graph, including SP (Sec/SPI) / LIPO (Sec/SPII) / TAT (Tat/SPI); indicate that the mutated signal peptide has improved the likelihood of the recombinant hGH secretion (Fig. 3). The secretory pathways of Sec and twin-arginine translocation (Tat) pathways function in parallel to transport proteins across the cytoplasmic membranes of prokaryotes as unfolded and folded, respectively (Palmer et al. 2012). So far, several proteins have been secreted extracellularly with this strategy using modified forms of signal peptides [23, 25–34].

In this study, a series of amino acid changes were made in different regions of the signal peptide, including N, H and C domains.to evaluate and compare the efficiency of signal peptides in directing the recombinant hGH into the periplasmic and extracellular space. These changes were able to increase the hydrophilicity of the N domain and vice versa the hydrophobicity of the H region and finally increased the efficiency of this signal and optimized it for the secretion of hGH through the Sec pathway. Analysis of the hydropathy plots and examination of their different domains from the perspective of hydrophobicity and hydrophilicity showed obvious differences in different domains. Because of these changes, for example, the replacement of arginine instead of leucine in the N region has created a more positive charge in this area which might facilitate membrane entry and possibly increased the ability of the signal peptide to enter a negatively charged membrane. Another important change in the H region is the replacement of the amino acid isoleucine instead of threonine and the amino acid serine with alanine in the hydrophobic region, which has also increased the hydrophobicity of the this domain. Although the changes resulted in the formation of the two amino acids arginine at positions 10 and 12 in the altered signal, its pattern does not match the known motif in the signal peptides of the Tat pathway, which is RRKR.

The search for various internal and foreign patents and the lack of similarity, highlights the innovation and the strength of our work. The cytoplasmic or periplasmic expression of recombinant hGH has many benefits, and therefore much research is being done in this field which has led to the identification of the mechanisms involved in protein secretion in microorganisms, pathways involved and different protein-protein interactions. Based on these findings, many efforts have been made to develop an efficient method for the secretion of recombinant proteins. One of the most important and effective approaches is the use of secretory signal peptides. Secretory signals play an important role in targeting proteins into the periplasmic and extracellular space [13, 14, 35–46] [47]. Since the mandatory addition of the amino acid methionine to protein for intracellular expression may elicit an immune response to the protein, this methionine must be removed in later steps. In addition, cytoplasmic production of hGH can lead to the formation of inclusion bodies in which proteins usually lack proper structure and function. Therefore, the secretion of hGH to the extracellular environment is the most appropriate method to obtain an active protein free of endotoxin.

We first tried to use the native SpA signal peptide, which has previously been used successfully in our laboratory to secrete a number of proteins. However, both bioinformatics analysis and experimental results showed that the native signal peptide is not effective in hGH secretion. The original peptide signal is based on protein A in the gram-positive *S. aureus*, but the altered signal peptide is redesigned for secretion or hGH in the gram-negative bacterium *E. coli*. However, the general secretion pathway in both signal peptides is through the sec pathway.

One of the challenges of using different signal peptides lies in the fact that a particular signal peptide of one protein may not necessarily function properly when fused to another protein. There is currently no specific method for finding an appropriate signal peptide for protein secretion and in many cases it is based on trial and error.

## Conclusions

In the present study, since the native signal protein peptide of *S. aureus* protein A was not able to deliver hGH to the extracellular space, it was modified using bioinformatics tools and fused to the n-terminal region of hGh to show that the redesigned signal peptide was functional. The efficiency of the redesigned signal peptide in the secretion of recombinant hGH into the periplasmic space as well as the extracellular environment was confirmed by evaluating the presence of hGH in different cell fractions.

Since the H and N domains in signal peptides play a key role in protein secretion, in this study, the hydrophobicity of the H region and the positive charge of the N region, which play an important role in cell membrane fusion, increased. Although the length of the H region has been shown to affect protein secretion and the length of the H region in gram-positive peptide signals is longer than in gram-negative signal peptides, in this study the peptide signal length did not change. A wide range of environmental factors affect gene expression and regulation.

Optimization of hGH production was performed using the RSM. To overcome the disadvantages of the classical experimental design method and evaluate various parameters with a smaller number of experiments, we performed RSM to optimize hGH expression and secretion. In RSM, input variables were changed to achieve the desired output [48–50]. We also defined some equations to maintain the best conditions. Determination of functional hGH concentration using a quantitative ELISA assay confirmed that the concentration of active form of hGH and its secretion in the periplasm is higher than the active form of hGH produced in the cytoplasm of bacterial cells. This demonstrates the importance of secreting recombinant pharmaceutical proteins to maintain their function. The results of optimization of hGH production showed that the factors of culture medium temperature, induction rate and time after induction have a positive effect on hGH production. It has also been shown that the use of glycine can improve hGH secretion in culture medium while the mechanism is not yet known.

Also in this study, using the appropriate algorithm and according to the eukaryotic origin of hGH gene, rare codons that reduce translation efficiency were changed in accordance with the *E. coli* expression system and its preferred codons to the highest possible level of gene expression. Provided that the CAI was upgraded from 0.85 to 0.87 by optimizing the codon. Meanwhile, GC content was also optimized to increase mRNA half-life and stability using related software. Stem-loop structures that prevent ribosomal binding to initiate translation and their stability were removed as much as possible.

Our results generally show that by increasing their efficiency, this prokaryotic secretory expression system could be used in the pharmaceutical industry to produce recombinant proteins. Although these experiments were performed in shake-flask cultures, the results of these experiments should also be performed in a fermenter to investigate the possibility of increasing the scale of protein production. Genetic manipulation of the host strain may also be required to make it more suitable for the expression and secretion of recombinant drug proteins.

## Materials and methods

### Bioinformatics analyzes

#### Gene optimization

Gene optimization was done for removing the structures that may inhibited high levels of protein expression in *E. coli* or ribosomal bonding, such as stem-loop structures, sequences cause mRNA instability, cis-acting elements and repetitive signals with negative effects on genes expression.

#### Investigating the efficiency of the secretory signal peptides

The secretory signal peptide was designed using the web-based bioinformatics tool (SignalP-version 5.0). The secretory signal peptide of the *S. aureus* protein A was modified by replacing different amino acids in its three different domains [42] and the ability of native and modified signal peptides in secretion of the hGH were investigated. The modified signal peptide was selected and evaluated by bioinformatics and several softwares such as SignalP-version 5.0, signal peptide (SP), tail anchor (TA) datasets, ProtScale., which mentioned in the text in the related sections.

#### mRNA stability analysis

To investigate the stability of mRNAs of the gene constructs, the sequences of the mRNAs were submitted to mfold software (http://unafold.rna.albany.edu/?q=mfold/RNA-Folding-Form) [16].

#### Tertiary structure analysis

The sequence of the optimized hGH gene was obtained from the Gene data bank with access code MT321110. Also, sequences of Spa native signal peptide from residues 1–36 (ID: TYO48081.1.) and mutant signal peptide (jei36c) (ID: QKG82153.1) were used in this study.

The tertiary structures of the hGH along with different attached signal peptides were predicted by submitting the sequences to the service IntFOLD (Version 5.0) [51, 52]. All of the structures were visualized by PyMOL (DeLano, et al. 2014) and the calculation procedure was as the same as that we reported in our previous works [53–56].

#### Gene cloning

Several recombinant structures were designed and constructed. In all constructs, the N-terminal Open Reading Frame region of the hGH was fused to an artificial secretory signal peptides based on the sec-dependent pathways signal peptides. *E. coli* (BL21 DE3) (Novagen, Darmstadt, Germany) competent cells were established by thermal shock as well as chemical treatment with calcium chloride and magnesium chloride. The competent cells were then juxtaposed to the recombinant structures. The cells were cultured in agar-LB culture medium [57] containing ampicillin 100 μg/ml and incubated at 37 °C for 16 h. The designed constructs containing the native and modified signal peptides fused to the coding region of the of the hGH were codon optimized and synthesized for expression in host *E. coli*.

#### Optimization of gene codons

The nucleotide sequence of the signal peptide was codon optimized (without changing the amino acid sequences) for expression in *E. coli.* The GenSmart™ Codon Optimization tools was used for the codon optimization. Optimization of gene codons was used to optimize a several factors that are critical for the efficiency of hGH gene expression in *E. coli* such as GC content, codon usage bias using codon adaptation index (CAI) and frequency of optimal codons (FOP) parameters.

#### Evaluation of recombinant protein expression in culture medium, periplasm and cytoplasm

SDS-PAGE and Western Blot analysis were used to evaluate the expression and secretion of the hGH in *E. coli* (BL21 DE3) cells. For this, 24 clones containing hGH gene were cultured separately in Luria-Bertani medium. After reaching the optical density (OD) of the culture medium at about 1.2–1.5, the medium was inoculated with 5% lactose and 0.5% glycine, and placed in a 30 °C. Sampling was done 5 and 16 h post inoculation. Expression of hGH in each of the 24 clones were investigated separately.

#### Fermentation of recombinant clones

A single colony from an *E. coli* BL21 DE3 carrying hGH gene was transferred to a 1000 ml flask containing 200 ml of F1 medium (Contents of F1culture medium are mentioned in Table 5) and incubated at 37 °C, 200 rpm for 16 h in an incubator shaker.

**Table 5:** Contents of F1culture medium

Materials	Glycerol	KH_2_pO_4_	K _2_HpO_4_	(NH_4_)_2_SO_4_	Yeast Extract	Peptone	MgSo_4_	Trace element
**Volume (g/l)**	20-40	9	6	0.5	5	5	1	1 (ml/l)

The New Brunswick fermenter (model *Bioflo 3* with a 4-l capacity reservoir) was used for the production of hGH. F1 medium components were prepared individually and sterilized prior to use. Prepared culture medium was inoculated with 200 ml pre-culture medium.

After inoculation, the fermenter operating conditions were set at 37 °C, 400 rpm stirrer and aeration of 1 l per minute. The starting pH was set at 7.0. After 4 h, lactose was added to the culture as an inducer and the process was continued with the same conditions for 10 h. The process was terminated before the consumption of the carbon source.

#### Protein expression confirmation using SDS-PAGE and Western blot analysis

*E. coli* BL21 (DE3) in LB medium with 50 μg/μl kanamycin (Merck, Germany) and 7% lactose (Merck, Germany) as an inducer was used for protein expression. Cells were collected and lysis buffer (50 mM Tris base, 10% glycerol, 0.1%Triton X-100) (Merck, Germany) was added to the cells. Total protein was extracted and analyzed using polyacrylamide gel. Transformed bacteria were induced by lactose and sampling was done in different time points T0, T5 and T overnight (To/n). At each stage, the bacteria were precipitated at (5000 rpm for 10 min) and the protein contents were precipitated by adding 100% TCA to the supernatant medium. The bacterial pellets were dissolved in the sample buffer as follows: T0 = in 120 μl sample buffer, T5 = in 250 μl sample buffer, To/n = in 350 μl sample buffer.

Resolved proteins were transferred from SDS-PAGE to the nitrocellulose paper (Wathman, UK). TBS buffer (Sigma, USA) was used for blocking the membrane. Polyclonal antibody produced against hGH was used to approve its expression. Anti-rabbit-HRP conjugated was used as a secondary antibody and the band were decrypted using addition of the 4-chloronaphthol substrate.

#### Protein purification

Total protein was extracted from the bacteria and hGH was purified using affinity chromatography by Ni-NTA Agarose (Qiagen, USA) based on the manufacturer’s instructions. Ni-NTA Agarose is an affinity chromatography matrix for purifying proteins including a 6XHis-tag. Histidine residues in the 6XHis-tag bind to the sites in the immobilized nickel ions with high specificity and affinity.

### Optimization of hGH expression

The effects of four variables including temperature, IPTG concentration, bacterial OD at the time of induction and the incubation time after induction) on the production of periplasmic and cytoplasmic recombinant hGh hormone in *E. coli* BL21 (DE3) were analyzed using RSM by a central composite design (CCD, Design-Expert v. 11; Stat-Ease, Minneapolis, MN, USA). The expression levels after induction were determined by eventuating the hGh concentrations of the cytoplasmic as well as periplasmic fractions. The amounts of the independent variables along with the corresponding levels used in the central composite design are shown in Table 2.

### Determination of functional hGH concentration using an ELISA-based method

In this experiment, the hGH quantitative test kit (Padtan Elm company, Iran) based on solid phase enzyme immunoassay (EIA) method contains two mouse monoclonal antibodies that identify antigenic markers on the surface of the active form of hGH and is able to form To distinguish active hGH from its inactive form, was used. Both standard hGH as well as our laboratory produced hGH were bound to the anti-hGH antibodies, result in developing a blue color. The intensity of the developed color is proportionate to the value of hGH in the sample. The optical density was measured in a 96-Well plate by ELISA reader at 450 nm. Standard curves were created for each test by planning the absorbance amount versus the concentration of each controls. The hGH concentrations of the samples were then taken from the standard curve.

## Supplementary Information

**Additional file 1.** The schematic view of the pET26 plasmid as well as the designed gene structure.

**Additional file 2.** The original, unprocessed and uncropped version of the Fig. 7 in manuscript.

**Additional file 3.** The original, unprocessed and uncropped version of the Fig. 8 in manuscript.

**Additional file 4.** The original, unprocessed and uncropped version of the Fig. 9 in manuscript.

**Additional file 5.** After generating the standard curves for each test by plotting the absorbance versus the concentration of each controls, concentrations of the active form of the hGH were obtained from the standard curve (all row data were reported in this supplementary data file).

## Data Availability

All data generated or analyzed during this study are included in this published article and its supplementary information files 2–5 and tables no: 1–5. Sequence data used in this study is deposited in the GeneBank under the accession Number MT321110.
