# Supplementary material for: Optimization of expression, purification and secretion of functional recombinant human growth hormone in Escherichia coli using modified staphylococcal protein a signal peptide
Source: BMC Biotechnol. 2021 Aug 16;21:51. doi: 10.1186/s12896-021-00701-x (PMC8369807; doi:10.1186/s12896-021-00701-x)
Supplement: Supplementary file 4 — Additional file 4. The original, unprocessed and uncropped version of the Fig. 9 in manuscript. [file 12896_2021_701_MOESM4_ESM.docx]

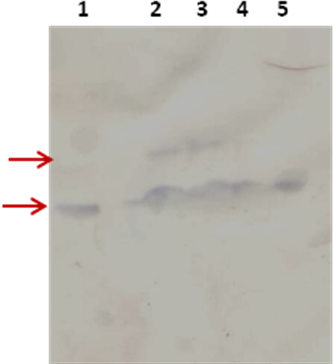


**Supplementary data file 4.** The original, unprocessed and uncropped version of the Fig. 9 in manuscript.
