# Supplementary material for: Optimization of expression, purification and secretion of functional recombinant human growth hormone in Escherichia coli using modified staphylococcal protein a signal peptide
Source: BMC Biotechnol. 2021 Aug 16;21:51. doi: 10.1186/s12896-021-00701-x (PMC8369807; doi:10.1186/s12896-021-00701-x)
Supplement: Supplementary file 5 — Additional file 5. After generating the standard curves for each test by plotting the absorbance versus the concentration of each controls, concentrations of the active form of the hGH were obtained from the standard curve (all row data were reported in this supplementary data file). [file 12896_2021_701_MOESM5_ESM.docx]

**Absorption of standard samples of the kit**

| [hGH] (ng/ml) | abs |
| --- | --- |
| 0 | 0 |
| 2.5 | 0.132 |
| 5 | 0.2 |
| 10 | 0.4 |
| 25 | 0.98 |
| 50 | 1.44 |

**Absorption of unknown protein samples**

| Fraction samples | Absorption at 450 nm | Protein concentration mg/ml |  |  |
| --- | --- | --- | --- | --- |
| Periplasmic sample 1/100000 dilution ratio | 0.06 | -0.58076 | -58075.6 |  |
| Periplasmic sample 1/100000 dilution ratio | 0.073 | -0.13402 | -13402.1 |  |
| Periplasmic sample 1/50000 dilution ratio | 0.093 | 0.553265 | 27663.23 | 0.027663 |
| Periplasmic sample 1/50000 dilution ratio | 0.103 | 0.896907 | 44845.36 | 0.044845 |
| Periplasmic sample 1/10000 dilution ratio | 0.329 | 8.66323 | 86632.3 | 0.086632 |
| Periplasmic sample 1/10000 dilution ratio | 0.371 | 10.10653 | 101065.3 | 0.101065 |
| Cytoplasmic sample 1/100000 dilution ratio | 0.04 | -1.26804 | -126804 |  |
| Cytoplasmic sample 1/100000 dilution ratio | 0.03 | -1.61168 | -161168 |  |
| Cytoplasmic sample 1/50000 dilution ratio | 0.075 | -0.06529 | -3264.6 | -0.00326 |
| Cytoplasmic sample 1/50000 dilution ratio | 0.082 | 0.175258 | 8762.887 | 0.008763 |
| Cytoplasmic sample 1/10000 dilution ratio | 0.24 | 5.604811 | 56048.11 | 0.056048 |
| Cytoplasmic sample 1/10000 dilution ratio | 0.22 | 4.917526 | 49175.26 | 0.049175 |

**Standard protein measurement curve**


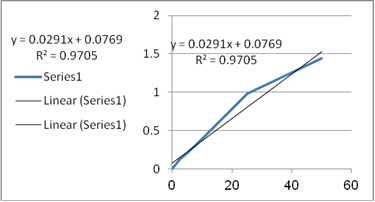


**Supplementary data file 2:** After generating the standard curves for each test by plotting the absorbance versus the concentration of each controls, concentrations of the active form of the hGH were obtained from the standard curve (all row data were reported in this supplementary data file)
